# ﻿Species diversity of *Pseudoplagiostoma* and *Pyrispora* (*Diaporthales*) from *Fagaceae* hosts in China

**DOI:** 10.3897/imafungus.16.153782

**Published:** 2025-05-23

**Authors:** Ning Jiang, Han Xue, Yong Li

**Affiliations:** 1 Key Laboratory of Forest Protection of National Forestry and Grassland Administration, Ecology and Nature Conservation Institute, Chinese Academy of Forestry, Beijing 100091, China Chinese Academy of Forestry Beijing China

**Keywords:** *Ascomycota*, molecular phylogeny, *Sordariomycetes*, systematics, taxonomy

## Abstract

*Diaporthales* is an important fungal order comprising plant-associated pathogens, endophytes, and saprobes in commercial crops and forest trees. Over the past decades, utilizing multiple gene phylogeny has substantially advanced our understanding of taxonomic relationships within this order, leading to the recognition of 35 morphologically and molecularly well-supported families. Among these, Pseudoplagiostoma (Pseudoplagiostomataceae) and Pyrispora (Pyrisporaceae) form two phylogenetically closely related lineages that exhibit distinct morphological characteristics. In this study, we conducted comprehensive morphological and phylogenetic analyses of fungal specimens associated with *Fagaceae* hosts and proposed four new species and two new combinations: *Ps.fagaceaearum***sp. nov.**, *Ps.neocastanopsidis***sp. nov.**, *Ps.quercus***sp. nov.**, *Py.humilis***comb. nov.**, *Py.myracrodruonis***comb. nov.**, and *Py.quercicola***sp. nov.** Furthermore, based on detailed morphological comparisons and molecular evidence, we synonymized *Neoplagiostoma* with *Pyrispora*, *Ps.castaneae* and *N.castaneae* with *Py.castaneae*, *Ps.ilicis* with *Ps.wuyishanense* and *Ps.diaoluoshanense* with *Ps.mangiferae*. This study provides substantial morphological and molecular data that significantly contribute to our understanding of *Pseudoplagiostomataceae* and *Pyrisporaceae*, thereby establishing a robust foundation for future taxonomic revisions and systematic investigations within *Diaporthales*. The findings not only expand our knowledge of fungal diversity associated with *Fagaceae* but also enhance our comprehension of evolutionary relationships within these important fungal families.

## ﻿Introduction

The fungal order *Diaporthales* represents a monophyletic group within Sordariomycetes (Ascomycota). Morphologically, members of *Diaporthales* are characterized by their teleomorphic features, which include solitary or aggregated, immersed or erumpent, orange, brown, or black perithecial ascomata in stromatic tissues or substrates, often with a defined centrum; unitunicate asci with a prominent refractive ring; short to elongate, aseptate or septate, hyaline or pigmented ascospores (Barr 1978; Crous et al. 2012a; Senanayake et al. 2017a, 2018). In contrast, the anamorphs within this order exhibit remarkable diversity, displaying acervular, pycnidial, or synnematal conidiomata, and produce unicellular to septate, hyaline to pigmented, various shaped conidia (Norphanphoun et al. 2016; Braun et al. 2018; Jiang et al. 2019a, 2021b, 2025; Jaklitsch and Voglmayr 2020; Cai et al. 2024). Ecologically, nearly all *Diaporthales* species are associated with plants, functioning as endophytes, pathogens, or saprobes (Crous et al. 2012b; Fan et al. 2018; Dissanayake et al. 2024). Notably, many of these fungi inhabit tree species, where they commonly cause diseases such as leaf spots, branch and twig cankers, and fruit rots (Lennox et al. 2004; Shuttleworth and Guest 2017; Jayawardena et al. 2018; Udayanga et al. 2021; Liu et al. 2024). Among these, the most renowned is *Cryphonectriaparasitica*, the causal agent of chestnut blight, which has had devastating effects on *Castaneadentata* populations (Jiang et al. 2019b, 2020). This pathogen exemplifies the significant ecological and economic impacts that *Diaporthales* fungi can have on forest ecosystems and plant health (Zhang and Blackwell 2001; Xavier et al. 2019; Roux et al. 2020; Jiang et al. 2023; Zauza et al. 2023; Zhu et al. 2024). Currently, *Diaporthales* contains 35 families supported by combined morphology and molecular phylogeny (Zhang et al. 2025).

The family *Pseudoplagiostomataceae*, containing a sole genus *Pseudoplagiostoma*, was originally established by Cheewangkoon et al. (2010) to accommodate three fungal species associated with *Eucalyptus* leaf diseases, with *Ps.eucalypti* designated as the type species. Subsequent taxonomic studies have expanded this genus, with most described species being plant pathogens predominantly distributed in subtropical and tropical regions (Suwannarach et al. 2016; Wang et al. 2016; Phookamsak et al. 2019; Silva et al. 2023; Zhang et al. 2023; Haituk et al. 2024; Mu et al. 2024a, 2024b; Wu et al. 2024; Zhang et al. 2025). Notably, *Ps.eucalypti* has been identified as an economically significant pathogen, causing black spot disease on *Eucalyptus* plantations in Guangxi, China and emerging as a destructive pathogen in northern India (Cao et al. 2023; Negi et al. 2024). Another important species, *Ps.mangiferae*, has been reported as a causal agent of mango leaf disease in Taiwan, China (Zhou et al. 2022).

The family *Pyrisporaceae*, currently comprising a single genus *Pyrispora* with its type species *P.castaneae*, was established based on a leaf-inhabiting fungus isolated from *Castaneamollissima* in China (Jiang et al. 2021a). This species exhibits diaporthalean characteristics in both teleomorphic and anamorphic stages, displaying morphological similarities with members of *Gnomoniaceae* (Jiang et al. 2021a). Nevertheless, comprehensive phylogenetic analyses have demonstrated that *Pyrisporaceae* represents a distinct evolutionary lineage separate from *Gnomoniaceae* (Jiang et al. 2021a). Interestingly, a morphologically similar fungus, *Ps.castaneae*, was subsequently described from *C.mollissima* in Shandong, China (Mu et al. 2022). However, morphological comparisons and molecular phylogenetic studies have revealed that this taxon represents a synonym of *Pyrisporacastaneae* (Mu et al. 2024a, 2024b).

Morphologically, members of *Pseudoplagiostomataceae* share several characteristics with *Gnomoniaceae* in their teleomorphic stage, particularly in possessing solitary, immersed, non-stromatic ascomata with lateral beaks, asci featuring distinct apical rings, and 1-septate ascospores (Barr 1978; Cheewangkoon et al. 2010). A distinctive feature of *Pseudoplagiostomataceae* is the production of thick-walled conidia, a characteristic also observed in *Plagiostoma* species within *Gnomoniaceae* (Senanayake et al. 2017a, 2018; Yang et al. 2020). In contrast, *Pyrisporaceae*, while also exhibiting non-stromatic ascomata, is distinguished by the presence of aseptate ascospores, a key diagnostic feature that differentiates it from *Pseudoplagiostomataceae* (Jiang et al. 2021a). Moreover, the absence of thick-walled conidia in *Pyrisporaceae* serves as a significant morphological character for distinguishing between these two taxa (Jiang et al. 2021a).

In this study, we investigated fungal pathogens associated with *Fagaceae*, a crucial plant family widely distributed in China. We specifically collected diseased leaf samples to isolate and identify fungal strains belonging to the genera *Pseudoplagiostoma* and *Pyrispora*. The primary objectives of this research are: (1) to elucidate the species diversity of these two diaporthalean genera associated with *Fagaceae* hosts, and (2) to clarify and refine the taxonomic concepts of *Pseudoplagiostoma* and *Pyrispora* through comprehensive morphological and molecular analyses.

## ﻿Materials and methods

### ﻿Surveys and isolations

Leaves of various *Fagaceae* hosts including *Castanopsiscarlesii*, *Ca.choboensis*, *Ca.patelliformis*, *Cyclobalanopsispatelliformis*, *Quercusaliena*, *Q.engleriana*, and *Q.variabilis* with leaf spots were collected across Anhui, Guizhou, Hainan, and Henan Provinces in China in 2019. The sampled leaves were transported to the laboratory in paper bags for fungal isolation. Initially, the leaves were rinsed with tap water to remove surface debris and dried using sterilized absorbent cotton. Subsequently, the samples were surface disinfected by immersing them in 95% ethanol for 10 seconds, 10% NaOCl for 2 minutes, 70% ethanol for 2 minutes, followed by rinsing in distilled water for 2 minutes and drying again with sterilized absorbent cotton. The leaves were then aseptically cut into 0.5 × 0.5 cm pieces using a sterile double-edged blade. Pieces containing both diseased and healthy tissues were transferred onto the potato dextrose agar (PDA; containing 200 g potatoes, 20 g dextrose, and 20 g agar per liter) and incubated at 25 °C to obtain pure fungal cultures. Dried cultures such as the fungarium specimens were deposited in the herbarium of the Chinese Academy of Forestry (CAF; http://museum.caf.ac.cn/), and the isolates were preserved at the China Forestry Culture Collection Center (CFCC; https://cfcc.caf.ac.cn/).

### ﻿Morphological analyses

The isolates obtained in this study were subcultured on PDA, malt extract agar (MEA; 30 g malt extract, 5 g mycological peptone, 15 g agar per liter), and synthetic nutrient agar (SNA; 0.2 g glucose, 0.2 g sucrose, 1 g potassium dihydrogen phosphate, 1 g potassium nitrate, 0.25 g magnesium sulfate anhydrous, 0.5 g potassium chloride, 14 g agar per liter) plates to induce the formation of fruiting bodies. Colony characteristics were observed and documented. Rayner (1970) was followed for colony color determination. Sporulated cultures were examined using a Zeiss Discovery V8 stereomicroscope (Jena, Germany) and microscopic structures, including conidiophores, conidiogenous cells, and conidia, were photographed using an Olympus BX51 microscope (Tokyo, Japan). A total of 30 conidiogenous cells and 50 conidia of each species were randomly selected for measurement, with the results presented as maximum and minimum values (in parentheses), along with the range expressed as the mean ± standard deviation.

### ﻿Molecular analyses

Fungal genomic DNA was extracted from colonies cultivated on PDA plates for 10 days using the Wizard® Genomic DNA Purification Kit (Promega, Madison, WI, USA), adhering to the manufacturer’s instructions. Five loci were targeted for amplification: the internal transcribed spacer (ITS) region, the large subunit nrDNA (LSU), the DNA-directed RNA polymerase II second largest subunit (*RPB2*), the translation elongation factor 1-alpha (*TEF1-α*), and the partial beta-tubulin (*TUB2*) genes. The primer pairs used for amplification were ITS1/ITS4 for ITS, LR0R/LR5 for LSU, RPB2-5F/fRPB2-7cR for *RPB2*, EF1-728F/EF1-986R or EF1-728F/EF2 for *TEF1-α*, and Bt2a/Bt2b for *TUB2* (Vilgalys and Hester 1990; White et al. 1990; Glass and Donaldson 1995; Carbone and Kohn 1999; Liu et al. 1999; Rehner et al. 2001). Polymerase chain reaction (PCR) was performed under the following conditions: initial denaturation at 94 °C for 5 min, followed by 35 cycles of denaturation at 94 °C for 30 sec, annealing at 48 °C (for ITS and LSU), 54 °C (for *TEF1-α* and *TUB2*), or 55 °C (for *RPB2*) for 50 sec, and extension at 72 °C for 1 min, with a final elongation step at 72 °C for 7 min. The resulting amplicons were sequenced bidirectionally using the same primers by Ruibo Xingke Biotechnology Company Limited (Beijing, China). The obtained sequences were assembled and edited using Seqman v. 7.1.0 (DNASTAR Inc., Madison, WI, USA) and subsequently deposited in the National Center for Biotechnology Information (NCBI) database (Suppl. material 1). Sequence alignments for the five loci (ITS, LSU, *RPB2*, *TEF1-α*, and *TUB2*) were conducted using MAFFT v. 7 (Katoh and Standley 2023) and further refined through manual adjustments in MEGA v. 7.0.21.

Phylogenetic analyses were performed on the concatenated dataset of the five loci using both Maximum Likelihood (ML) and Bayesian Inference (BI) approaches. For the ML analysis, the GTR substitution model was employed, and 1000 bootstrap replicates were conducted through the CIPRES Science Gateway portal (https://www.phylo.org/; Miller et al. 2010) using RAxML-HPC BlackBox v. 8.2.10 (Stamatakis 2008). For the BI analysis, partition-specific evolutionary models were selected using MrModeltest v. 2.3 based on the Akaike Information Criterion (AIC). Markov Chain Monte Carlo (MCMC) simulations were executed in MrBayes v. 3.1.2 (Ronquist and Huelsenbeck 2003) with two independent runs, each comprising 10 million generations and starting from random trees. Convergence of the runs was confirmed by ensuring the average standard deviation of split frequencies was below 0.01, and trees were sampled every 1000 generations. The initial 25% of the sampled trees were discarded as burn-in, and posterior probabilities (BPP) were calculated from the remaining trees. Bootstrap support (BS) values in the ML analysis were derived from 1000 replicates, and the resulting phylogenetic trees were visualized and annotated using FigTree v. 1.4.4 (Rambaut 2018).

The pairwise homoplasy index (PHI) test was performed using the SplitsTree App to assess potential recombination events among closely related phylogenetic species (Huson and Bryant 2024). The analysis utilized a concatenated dataset of five loci (ITS, LSU, *RPB2*, *TEF1-α* and *TUB2*), and the Φw-statistic below 0.05 (*p*-value < 0.05) revealed significant evidence of recombination. To further elucidate relationships among closely related taxa, split graphs were constructed using the Log-Det transformation and split decomposition methods, offering a clear and intuitive visualization of phylogenetic associations.

## ﻿Results

### ﻿Phylogeny

**Figure 1.**
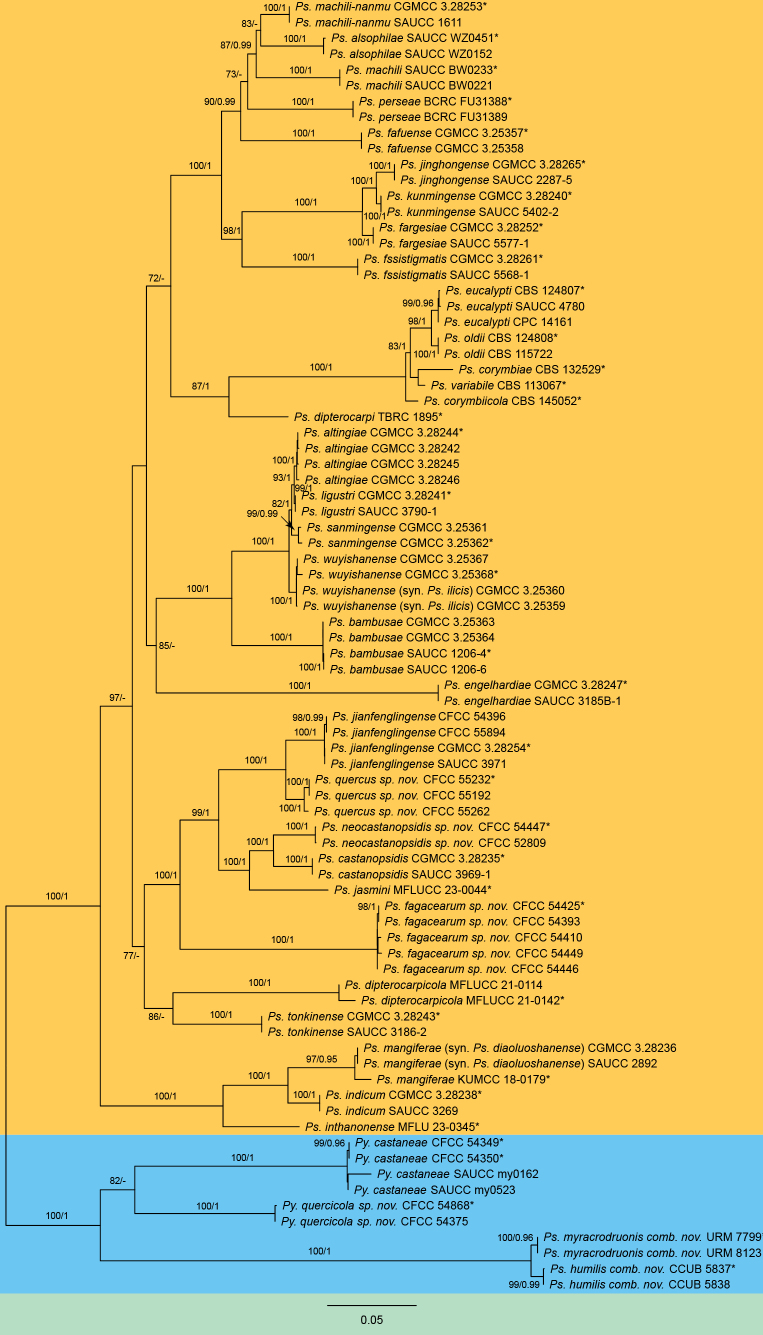
Phylogram of *Pseudoplagiostoma* and *Pyrispora* resulting from a maximum likelihood analysis based on the ITS, LSU, *RPB2*, *TEF1-α* and *TUB2* gene loci. Numbers above the branches indicate ML bootstrap values (left, MLBS ≥ 70%) and Bayesian posterior probabilities (right, BPP ≥ 0.95). Ex-type strains are marked with *.

The concatenated dataset of ITS, LSU, *RPB2*, *TEF1-α*, and *TUB2* included 82 strains, encompassing a total of 3,230 characters (ITS: 1-553; LSU: 554-1,349; *RPB2*: 1,350-2,207; *TEF1-α*: 2,208-2,720; *TUB2*: 2,721-3,230), with gaps included. The maximum likelihood (ML) analysis yielded an optimization likelihood value of -24659.30 for the best RAxML tree, with the alignment matrix containing 1,328 distinct patterns and 20.21% undetermined characters or gaps. The estimated nucleotide frequencies were as follows: A = 0.232135, C = 0.269862, G = 0.265440, and T = 0.232563. The substitution rates were calculated as AC = 2.097626, AG = 4.485069, AT = 1.996030, CG = 1.192343, CT = 8.147705, and GT = 1.0. The gamma distribution shape parameter (α) was estimated at 0.220442. For Bayesian inference (BI) analysis, the most suitable evolutionary models for each locus were determined using MrModeltest, with SYM+I+G4 selected for ITS, TNe+R2 for LSU, TIM3e+I+G4 for *RPB2*, TPM2+F+I+G4 for *TEF1-α*, and HKY+F+I+G4 for *TUB2*. The BI results were consistent with the ML tree topology. Branches in Fig. 1 are annotated with ML bootstrap support values (BS) ≥ 70% and Bayesian posterior probabilities (BPP) ≥ 0.95. Phylogenetic analysis revealed that the 14 isolates formed five well-defined clades within *Pseudoplagiostoma* and *Pyrispora*, representing four novel species and a known species (Fig. 1). Notably, *Ps.wuyishanense* and *Ps.ilicis* form a single clade with high support values (MLB/PP = 100/1), while *Ps.diaoluoshanense* and *Ps.mangiferae* form another well-supported distinct clade (MLB/PP = 97/0.95). In addition, isolates of *Ps.humilis* and *Ps.myracrodruonis* clustered as two supported clades within *Pyrispora*.

### ﻿PHI analysis

**Figure 2.**
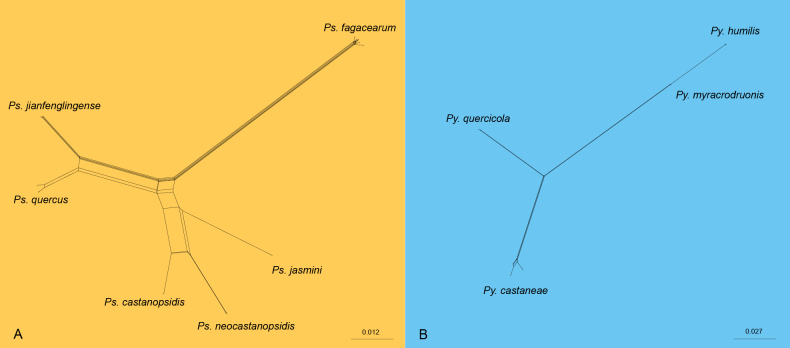
The split graphs of a PHI test result of *Pseudoplagiostoma* and *Pyrispora* species using the LogDet transformation and split decomposition options based on the combined ITS, LSU, *RPB2*, *TEF1-α* and *TUB2* gene loci. **A***p* = 1.0. **B***p* = 1.0.

To validate species delineation within *Pseudoplagiostoma* and *Pyrispora*, PHI analysis was conducted. Two clades, comprising both established and newly proposed species, were selected for testing: Clade A included *Ps.castanopsidis*, *Ps.jasmini*, *Ps.jianfenglingense*, *Ps.fagacearum*, *Ps.neocastanopsidis*, and *Ps.quercus*, while Clade B comprised *Py.castaneae*, *Py.humilis*, *Py.myracrodruonis*, and *Py.quercicola*. The PHI test revealed no significant evidence of genetic recombination within these clades (Clade A, *p* = 1.0; Clade B, *p* = 1.0; Fig. 2). These results provide robust support for the genetic distinctness of the four new species proposed in this study, confirming their validity as separate taxonomic entities.

### ﻿Taxonomy

#### ﻿ Pseudoplagiostoma fagacearum

Taxon classificationAnimaliaDiaporthalesFagaceae

Ning Jiang sp. nov.

024F62F4-F909-59DA-851B-E4CEB5CFF92F

841327

Fig. 3

##### Etymology.

Named after the host family, *Fagaceae*.

##### Diagnosis.

Distinct from *Ps.castanopsidis* by longer conidia; and from *Ps.jasmini*, *Ps.jianfenglingense*, *Ps.neocastanopsidis* and *Ps.quercus* by wider conidia.

**Figure 3.**
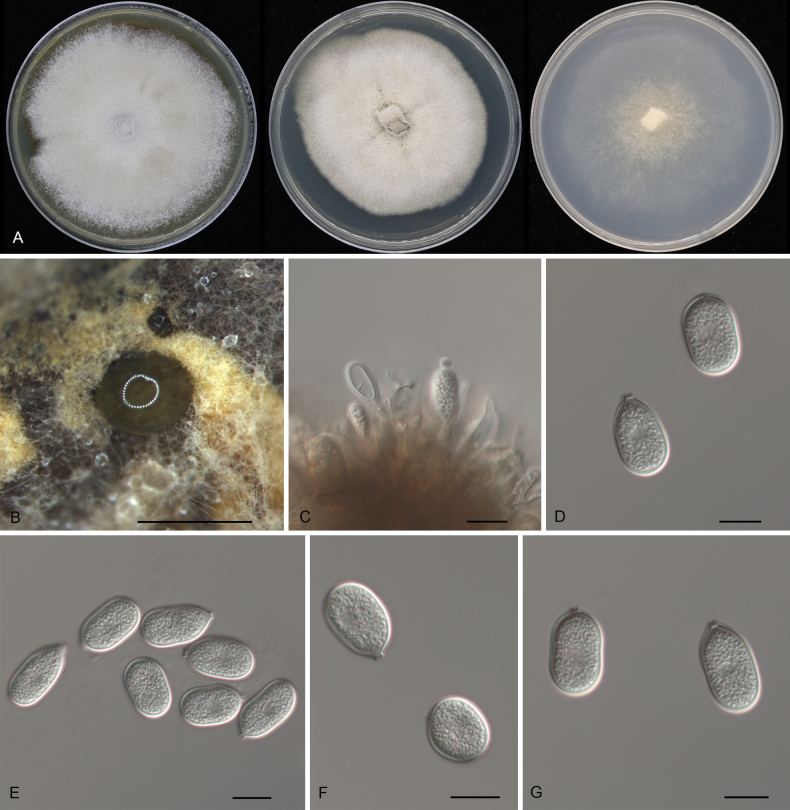
Morphology of *Pseudoplagiostomafagacearum* (CFCC 54425): **A** colonies on PDA, MEA and SNA after 10 days at 25 °C **B** conidioma formed on PDA**C** conidiogenous cells giving rise to conidia **D–G** conidia. Scale bars: 500 μm (**B**); 10 μm (**C–G**).

##### Typus.

CHINA • Guizhou Province, Zunyi City, Suiyang County, Kuankuoshui Natural Reserve, on diseased leaves of *Quercusengleriana*, 23 November 2019, Dan-ran Bian (**holotype** CAF800039; ex-type culture CFCC 54425).

##### Description.

***Conidiomata*** pycnidial, solitary, erumpent, globose to pulvinate, brown, 400–750 μm diam., exuding a brown conidial mass. ***Conidiophores*** indistinct, often reduced to conidiogenous cells. ***Conidiogenous cells*** hyaline, smooth, lageniform to ampulliform, attenuate towards the apex, phialidic, 13.5–18.5 × 4–5.5 μm. ***Conidia*** holoblastic, aseptate, hyaline, smooth, thick-walled, multi-guttulate, ellipsoid, oblong-cylindrical, slightly constricted at the middle, slightly curved, base tapering to a flat protruding scar, (18–)18.5–21(–22.5) × (10.5–)11–13(–14) μm (n = 50), L/W = 1.4–2.1, with a prominent hilum.

##### Culture characteristics.

***Colonies*** on PDA flat, spreading, with flocculent aerial mycelia and floccose margin, vinaceous buff, reaching 90 mm diam. after 1 week at 25 °C, forming brown conidiomata with brown conidial masses. ***Colonies*** on MEA flat, dense, surface folded, with moderate flocculent aerial mycelia and even margin, lavender gray to buff, reaching 70 mm diam. after 2 weeks at 25 °C, sterile. ***Colonies*** on SNA flat, spreading, with sparse aerial mycelia and smooth margin, white to rosy buff, reaching 70 mm diam. after 2 weeks at 25 °C, sterile.

##### Additional materials examined.

CHINA • Guizhou Province, Zunyi City, Suiyang County, Kuankuoshui Natural Reserve, on diseased leaves of *Quercusengleriana*, 23 November 2019, Dan-ran Bian (cultures CFCC 54446 and CFCC 54410); • Guizhou Province, Zunyi City, Honghuagang District, Zunyi Normal University, on diseased leaves of *Castanopsischoboensis*, 24 November 2019, Shang Sun (culture CFCC 54449); • Hainan Province, Changjiang Li Autonomous County, Bawangling National Forest Park, on diseased leaves of *Cyclobalanopsispatelliformis*, 30 March 2019, Yong Li (culture CFCC 54393).

##### Distribution.

China, Guizhou and Hainan Provinces.

##### Ecology.

Associated with leaf spot disease of *Castanopsischoboensis*, *Cyclobalanopsispatelliformis* and *Quercusengleriana*.

##### Notes.

Five isolates obtained from leaf spots of *Castanopsischoboensis*, *Cyclobalanopsispatelliformis*, and *Quercusengleriana* formed a well-supported clade, which is newly described here as *Pseudoplagiostomafagacearum*. This species is phylogenetically closely related to *Ps.castanopsidis*, *Ps.jasmini*, *Ps.jianfenglingense*, *Ps.neocastanopsidis* and *Ps.quercus* (Fig. 1). However, *Ps.fagacearum* (18.5–21 × 11–13 μm) has wider conidia than *Ps.jasmini* (14–22 × 6.5–11 μm), *Ps.jianfenglingense* (19–22 × 8.5–11 μm), *Ps.neocastanopsidis* (19–22 × 9–10 μm), and *Ps.quercus* (17–21 × 9.5–11 μm), and longer conidia than *Ps.castanopsidis* (16.5–19.8 × 8.7–12.8 μm) (Gomdola et al. 2023; Zhang et al. 2025).

#### ﻿ Pseudoplagiostoma jianfenglingense

Taxon classificationAnimaliaDiaporthalesFagaceae

Zhao X. Zhang & X.G. Zhang, Fungal Diversity: 10.1007/s13225-025-00551-4 (2025)

E59C3897-E4B7-523A-B9C2-314401101EBE

Fig. 4

##### Description.

*Conidiomata* pycnidial, solitary, erumpent, globose, dark brown, 150–400 μm diam., exuding a dark conidial mass. *Conidiophores* indistinct, often reduced to conidiogenous cells. *Conidiogenous cells* hyaline, smooth, lageniform to ampulliform, phialidic, 11–26 × 4–6.5 μm. *Conidia* holoblastic, aseptate, hyaline, smooth, thick-walled, multi-guttulate, ellipsoid, oblong-cylindrical, slightly constricted at the middle, slightly curved, base tapering to a flat protruding scar, (16.5–)17.5–19.5(–20.5) × (10.5–)11–12.5(–13) μm (n = 50), L/W = 1.4–1.8, with a prominent hilum.

**Figure 4.**
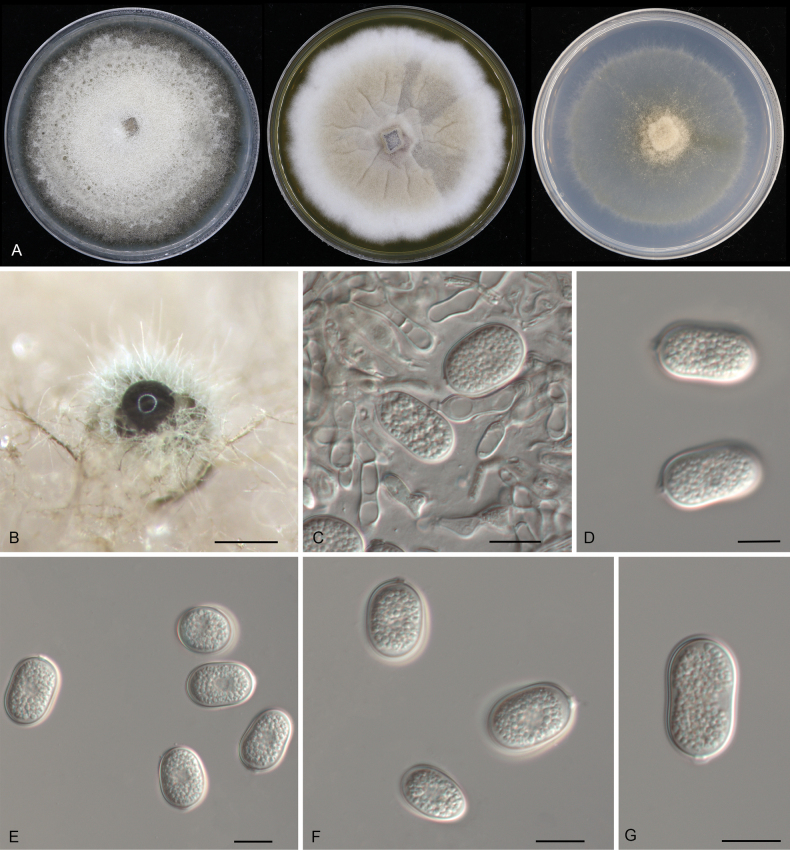
Morphology of *Pseudoplagiostomajianfenglingense* (CFCC 54396): **A** colonies on PDA, MEA and SNA after 10 days at 25 °C **B** conidioma formed on PDA**C** conidiogenous cells giving rise to conidia **D–G** conidia. Scale bars: 300 μm (**B**); 10 μm (**C–G**).

##### Culture characteristics.

***Colonies*** on PDA flat, spreading, with flocculent aerial mycelia and even margin, forming white to smoke grey circular center area and sepia outer area, fast growing, reaching 90 mm diam. after 1 week at 25 °C, forming dark brown conidiomata with dark conidial masses. ***Colonies*** on MEA flat, dense, surface folded, with abundant aerial mycelia and floccose margin, forming ochreous circular center area and white outer area, reaching 80 mm diam. after 2 weeks at 25 °C, sterile. ***Colonies*** on SNA flat, spreading, with sparse aerial mycelia and undulating margin, cinnamon, slowly growing, reaching 70 mm diam. after 3 weeks at 25 °C, sterile.

##### Materials examined.

CHINA • Hainan Province, Changjiang Li Autonomous County, Bawangling National Forest Park, on diseased leaves of *Cyclobalanopsispatelliformis*, 30 March 2019, Yong Li (CAF800038; cultures CFCC 54396 and CFCC 55894).

##### Distribution.

China, Hainan Province.

##### Ecology.

Associated with leaf spot disease of *Cyclobalanopsispatelliformis*.

##### Notes.

Two isolates obtained from leaf spots of *Cyclobalanopsispatelliformis* formed a well-supported clade with two isolates of *Pseudoplagiostomajianfenglingense* from unknown leaves (Fig. 1; Zhang et al. 2025). Hence, *Cy.patelliformis* become a new host for *Ps.jianfenglingense*.

#### ﻿ Pseudoplagiostoma mangiferae

Taxon classificationAnimaliaDiaporthalesFagaceae

Dayar., Phookamsak & K.D. Hyde, Fungal Diversity 95: 121 (2019)

8D6A183D-53EA-5ED4-8FE0-D57E0F02EDE7

##### Synonym.

*Pseudoplagiostomadiaoluoshanense* Zhao X. Zhang & X.G. Zhang

##### Description.

See Phookamsak et al. (2019).

##### Distribution.

China, Hainan and Yunnan Provinces.

##### Ecology.

Associated with leaf blight disease of *Mangifera* hosts.

##### Notes.

*Pseudoplagiostomamangiferae* was first described from Yunnan Province, China, where it was found associated with leaf blight symptoms on *Mangifera* sp. (Phookamsak et al. 2019). Subsequently, *Ps.diaoluoshanense* was proposed based on two isolates obtained from leaf spots of *Mangiferaindica* in Hainan, China (Zhang et al. 2025). Phylogenetic analysis revealed that these three isolates from *Mangifera* hosts formed a well-supported monophyletic clade (Fig. 1). Notably, they exhibit only minor sequence variations, with 2 bp differences in the ITS region and no variation in the LSU region.

#### ﻿ Pseudoplagiostoma neocastanopsidis

Taxon classificationAnimaliaDiaporthalesFagaceae

Ning Jiang sp. nov.

55AA6A4F-4C30-5FF0-AD7A-DA2B1251DBB1

841324

Fig. 5

##### Etymology.

Name refers to its closest relative, *Pseudoplagiostomacastanopsidis*.

**Figure 5.**
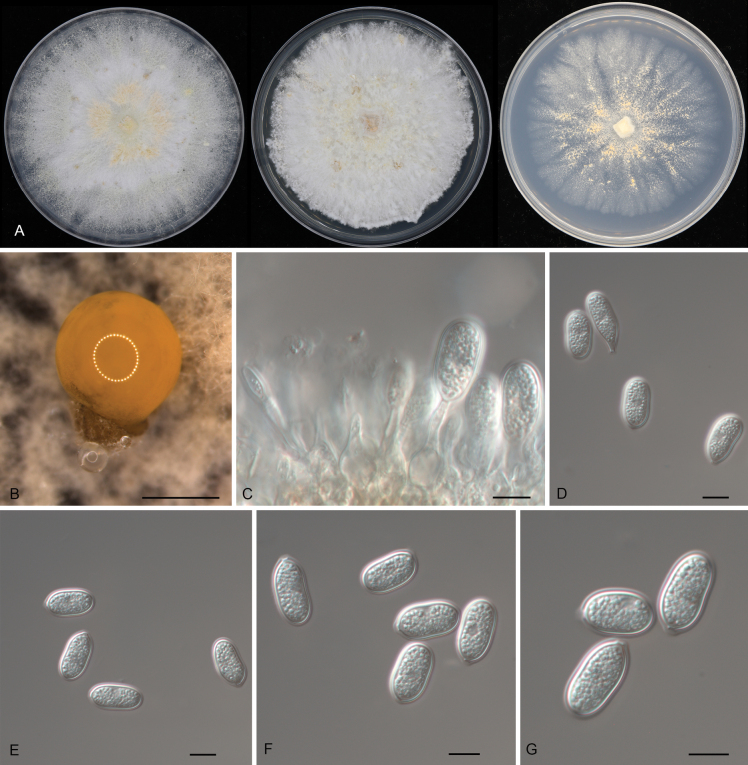
Morphology of *Pseudoplagiostomaneocastanopsidis* (CFCC 54447): **A** colonies on PDA, MEA and SNA after 10 days at 25 °C **B** conidioma formed on PDA**C** conidiogenous cells giving rise to conidia **D–G** conidia. Scale bars: 500 μm (**B**); 10 μm (**C–G**).

##### Diagnosis.

Distinct from its phylogenetically related species of *Ps.castanopsidis* and *Ps.jasmini* by wider conidiogenous cells.

##### Typus.

CHINA • Hainan Province, Changjiang Li Autonomous County, Bawangling National Forest Park, on diseased leaves of *Castanopsiscarlesii*, 30 March 2019, Yong Li (**holotype** CAF800037; ex-type culture CFCC 54447).

##### Description.

***Conidiomata*** pycnidial, solitary, erumpent, globose to pulvinate, brown, 300–700 μm diam., exuding an orange conidial mass. ***Conidiophores*** indistinct, often reduced to conidiogenous cells. ***Conidiogenous cells*** hyaline, smooth, lageniform to ampulliform, attenuate towards the apex, phialidic, 11.5–22 × 3.5–6.5 μm. ***Conidia*** holoblastic, aseptate, hyaline, smooth, thick-walled, multi-guttulate, ellipsoid, oblong-cylindrical, slightly constricted at the middle, slightly curved, base tapering to a flat protruding scar, (18.5–)19–22(–24) × (8.5–)9–10(–10.5) μm (n = 50), L/W = 1.8–2.7, with a prominent hilum.

##### Culture characteristics.

***Colonies*** on PDA flat, spreading, with abundant aerial mycelia and even margin, forming pale luteous center area, white middle area and smoke grey outer area, fast growing, reaching 90 mm diam. after 1 week at 25 °C, forming brown conidiomata with orange conidial masses. ***Colonies*** on MEA flat, spreading, with flocculent aerial mycelia and undulating margin, white to saffron, fast growing, reaching 90 mm diam. after 1 week at 25 °C, sterile. ***Colonies*** on SNA flat, spreading, with sparse aerial mycelia and feathery margin, pale luteous to orange, slowly growing, reaching 70 mm diam. after 3 weeks at 25 °C, sterile.

##### Additional material examined.

CHINA • Hainan Province, Changjiang Li Autonomous County, Bawangling National Forest Park, on diseased leaves of *Castanopsiscarlesii*, 30 March 2019, Yong Li (culture CFCC 52809).

##### Distribution.

China, Hainan Province.

##### Ecology.

Associated with leaf spot disease of *Castanopsiscarlesii*.

##### Notes.

Two isolates from leaf spots of *Castanopsiscarlesii* clustered in a well-supported clade here newly described as *Pseudoplagiostomaneocastanopsidis*, which is phylogenetically close to *Ps.castanopsidis* and *Ps.jasmini* (Fig. 1). Morphologically, *Ps.neocastanopsidis* is similar to *Ps.castanopsidis* and *Ps.jasmini* in conidial size (Gomdola et al. 2023; Zhang et al. 2025). However, *Ps.neocastanopsidis* differs from *Ps.jasmini* in conidial color (hyaline conidia in *Ps.neocastanopsidis* vs. brown conidia in *Ps.jasmini*). Furthermore, *Ps.neocastanopsidis* has wider conidiogenous cells than *Ps.castanopsidis* and *Ps.jasmini* (11.5–22 × 3.5–6.5 μm in *Ps.neocastanopsidis* vs. 9–16.8 × 2.2–2.9 μm in *Ps.castanopsidis* vs. 7.7–13.7 × 1.6–2.4 μm in *Ps.jasmini*) (Gomdola et al. 2023; Zhang et al. 2025).

#### ﻿ Pseudoplagiostoma quercus

Taxon classificationAnimaliaDiaporthalesFagaceae

Ning Jiang sp. nov.

D55E3A0D-833E-5859-ADEA-B346B1AFCC1E

841328

Fig. 6

##### Etymology.

Named after the host genus, *Quercus*.

**Figure 6.**
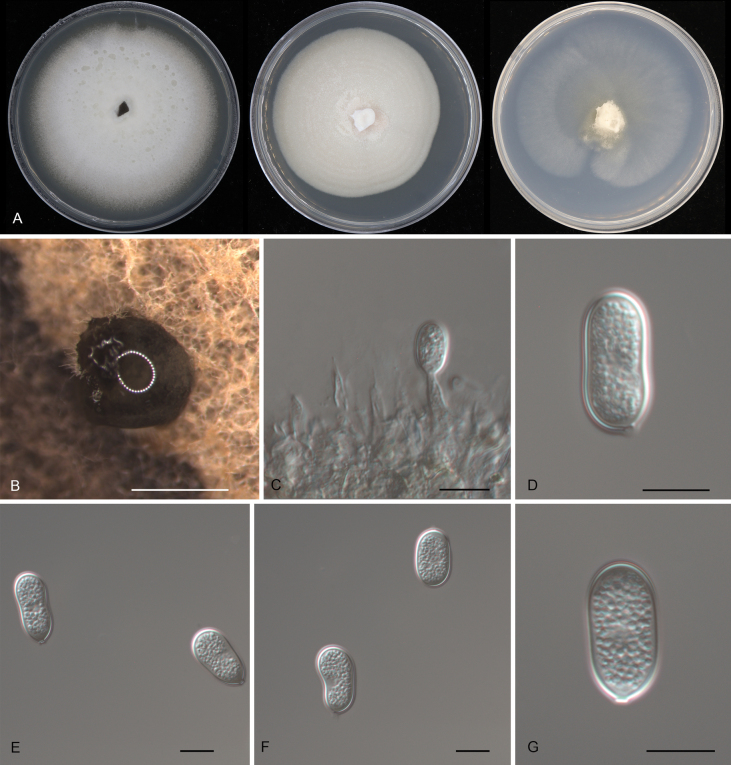
Morphology of *Pseudoplagiostomaquercus* (CFCC 55232): **A** colonies on PDA, MEA and SNA after 10 days at 25 °C **B** conidioma formed on PDA**C** conidiogenous cells giving rise to a conidium **D–G** conidia. Scale bars: 500 μm (**B**); 10 μm (**C–G**).

##### Diagnosis.

Distinct from its closely related species of *Ps.jianfenglingense* by sequence data.

##### Typus.

CHINA • Henan Province, Xinyang City, Pingqiao District, Haotang Village, on diseased leaves of *Quercusaliena*, 7 August 2019, Yong Li (**holotype** CAF800040; ex-type culture CFCC 55232).

##### Description.

***Conidiomata*** pycnidial, solitary, erumpent, globose to pulvinate, black, 300–700 μm diam., exuding a brown conidial mass. ***Conidiophores*** indistinct, often reduced to conidiogenous cells. ***Conidiogenous cells*** hyaline, smooth, ampulliform, attenuate towards the apex, phialidic, 10–25 × 2–8.5 μm. ***Conidia*** holoblastic, aseptate, hyaline, smooth, thick-walled, multi-guttulate, ellipsoid, oblong-cylindrical, slightly constricted at the middle, slightly curved, base tapering to a flat protruding scar, (16–)17–21(–21.5) × (8.5–)9.5–11(–11.5) μm (n = 50), L/W = 1.6–2.3, with a prominent hilum.

##### Culture characteristics.

***Colonies*** on PDA flat, spreading, with moderate aerial mycelia and smooth margin, white to smoke gray, reaching 80 mm. diam after 2 weeks at 25 °C, forming black conidiomata with brown conidial masses. ***Colonies*** on MEA flat, spreading, with sparse aerial mycelia and smooth margin, forming concentric rings, white to rosy buff, reaching 70 mm diam. after 2 weeks at 25 °C, sterile. ***Colonies*** on SNA flat, spreading, with sparse aerial mycelia and undulating margin, white to isabelline, slowly growing, reaching 70 mm diam. after 3 weeks at 25 °C, sterile.

##### Additional materials examined.

CHINA • Henan Province, Xinyang City, Pingqiao District, Haotang Village, on diseased leaves of *Quercusaliena*, 7 August 2019, Yong Li (culture CFCC 55192); • Henan Province, Xinyang City, Shihe District, Boerdeng Forest Park, on diseased leaves of *Quercusvariabilis*, 7 August 2019, Yong Li (culture CFCC 55262).

##### Distribution.

China, Henan Province.

##### Ecology.

Associated with leaf spot disease of *Quercusaliena* and *Q.variabilis*.

##### Notes.

Three isolates obtained from leaf spots of *Quercusaliena* and *Q.variabilis* formed a well-supported clade, described here as *Pseudoplagiostomaquercus*, which is phylogenetically sister to *Ps.jianfenglingense* (Fig. 1). Morphologically, *Ps.quercus* and *Ps.jianfenglingense* exhibit similar conidial shapes and dimensions. However, the two species can be clearly distinguished by sequence divergence, with nucleotide differences of 6/551 in ITS, 1/796 in LSU, 45/536 in *TEF-1α*, and 22/471 in *TUB2*.

#### ﻿ Pseudoplagiostoma wuyishanense

Taxon classificationAnimaliaDiaporthalesFagaceae

T.C. Mu & J. Zhi Qiu, J. Fungi 10(6, no. 383): 11 (2024)

33FD62E9-74B2-5C80-9861-2D52637CBB3F

##### Synonym.

*Pseudoplagiostomailicis* T.C. Mu & J.Z. Qiu

##### Description.

See Mu et al. (2024a, 2024b).

##### Distribution.

China.

##### Ecology.

Associated with leaf diseases of *Ilexchinensis*.

##### Notes.

*Pseudoplagiostomawuyishanense* was described as a novel species inhabiting branches of an unidentified tree in Fujian Province, China, based on morphological and phylogenetic analyses (Mu et al. 2024a). Subsequently, *Ps.ilicis* was proposed as a distinct species based on two isolates obtained from diseased leaves of *Ilexchinensis* in the same geographical region (Mu et al. 2024b). However, during the establishment of *P.ilicis*, no comparative analysis was conducted with the previously described *P.wuyishanense*. Through detailed examination, it has been determined that these two taxa exhibit identical morphological characteristics and phylogenetic positions (Fig. 1; Mu et al. 2024a, b). Consequently, in accordance with the principle of priority in taxonomic nomenclature based on publication dates, *Ps.ilicis* is hereby designated as a synonym of *Ps.wuyishanense*.

#### ﻿ Pyrispora

Taxon classificationAnimaliaDiaporthalesFagaceae

C.M. Tian & N. Jiang, J. Fungi 7(1, no. 64): 32 (2021)

AA1A379C-F43C-5BE5-9601-CB7EC2F9B905

##### Synonym.

*Neoplagiostoma* Z.X. Zhang & X.G. Zhang

##### Notes.

The genus *Pyrispora* was introduced with *Py.castaneae* as its type species, isolated from *Castaneamollissima* in China (Jiang et al. 2021a). Later, *Pseudoplagiostomacastaneae* was described from the same host and was subsequently treated as a synonym of *Py.castaneae* (Mu et al. 2022, 2024b). Recently, Zhang et al. (2025) proposed the genus *Neoplagiostoma*, typified by *N.castaneae* based on the basionym *Ps.castaneae*, but failed to compare it with *Pyrispora* either morphologically or phylogenetically. Our study confirms that *N.castaneae* and *Py.castaneae* are identical in host association, geographic distribution, morphology, and phylogenetic placement (Fig. 1). Therefore, *Neoplagiostoma* should be regarded as a synonym of *Pyrispora*.

#### ﻿ Pyrispora castaneae

Taxon classificationAnimaliaDiaporthalesFagaceae

C.M. Tian & N. Jiang, J. Fungi 7(1, no. 64): 32 (2021)

B2C492DB-49F3-5E21-BF7C-FECEA5D1B7C2

##### Synonyms.

*Neoplagiostomacastaneae* Z.X. Zhang & X.G. Zhang.

*Pseudoplagiostomacastaneae* T.C. Mu, J.W. Xia & X.G. Zhang.

##### Description.

See Jiang et al. (2021a).

##### Distribution.

China.

##### Ecology.

Associated with leaf diseases of *Castaneamollissima*.

##### Notes.

Based on the evidence presented above, *Neoplagiostomacastaneae* and *Pseudoplagiostomacastaneae* should be reduced to synonyms under *Pyrisporacastaneae*.

#### ﻿ Pyrispora humilis

Taxon classificationAnimaliaDiaporthalesFagaceae

(L.P.P. Magalhães, N.L.P. Sales & A. C. da Silva) Ning Jiang comb. nov.

CA0AF858-0021-51E4-BD26-16AB00C87112

858921

##### Basionym.

*Pseudoplagiostomahumilis* L.P.P. Magalhães, N.L.P. Sales & A. C. da Silva

##### Description.

See Magalhães et al. (2024).

##### Distribution.

Brazil.

##### Ecology.

Causing shoot blight and dieback of *Anacardiumhumile*.

##### Notes.

The genus *Pseudoplagiostoma* is characterized by producing holoblastic, hyaline to brown, ellipsoid, unicellular, subglobose to broadly allantoid, thick-walled conidia with a prominent hilum. Although *Ps.humilis* was recently described in this genus based on similar anamorphic characteristics (Magalhães et al. 2024), it differs notably by lacking both thick-walled conidia and a prominent hilum. Furthermore, our phylogenetic analyses robustly support the placement of this species within *Pyrispora* (Fig. 1). Based on these combined morphological and molecular evidences, we formally propose the transfer of *Ps.humilis* to *Pyrispora*.

#### ﻿ Pyrispora myracrodruonis

Taxon classificationAnimaliaDiaporthalesFagaceae

(A.P.S.L. Pádua, T.G.L. Oliveira, Souza-Motta, & J.D.P. Bezerra) Ning Jiang comb. nov.

F1B1B85D-ECE4-526F-A895-580D55DF00FF

857270

##### Basionym.

*Pseudoplagiostomamyracrodruonis* A.P.S.L. Pádua, T.G.L. Oliveira, Souza-Motta, & J.D.P. Bezerra

##### Description.

See Bezerra et al. (2019).

##### Distribution.

Brazil.

##### Ecology.

Endophytic in leaves of *Myracrodruonurundeuva*.

##### Notes.

Based on the same phylogenetic and morphological evidence presented above, we propose the transfer of *Pseudoplagiostomamyracrodruonis* to the genus *Pyrispora* as *Py.myracrodruonis* comb. nov.

#### ﻿ Pyrispora quercicola

Taxon classificationAnimaliaDiaporthalesFagaceae

Ning Jiang sp. nov.

6D56935B-F3A7-5851-91C5-56A1B85A0EAE

841329

Fig. 7

##### Etymology.

Named after the host genus *Quercus* and “-*cola*” = “inhabiting”.

**Figure 7.**
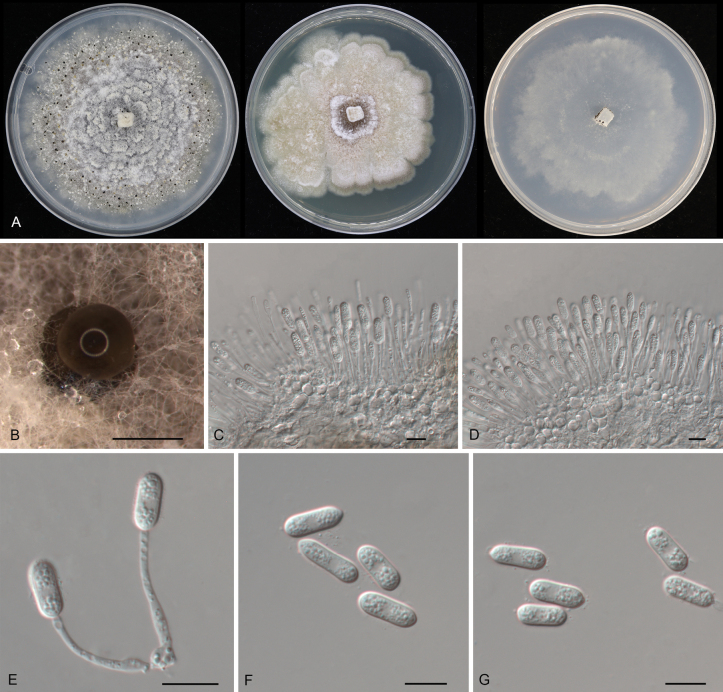
Morphology of *Pyrisporaquercicola* (CFCC 54868): **A** colonies on PDA, MEA and SNA after 10 days at 25 °C **B** conidioma formed on PDA**C–E** conidiogenous cells giving rise to conidia **F, G** conidia. Scale bars: 300 μm (**B**); 10 μm (**C–G**).

##### Diagnosis.

Distinct from *Py.castaneae* by the host genus.

##### Typus.

China • Anhui Province, Hefei City, Shushan District, Dashushan Forest Park, on diseased leaves of *Quercusaliena*, 2 November 2019, Dan-ran Bian (**holotype** CAF800041; ex-type culture CFCC 54868).

##### Description.

***Conidiomata*** pycnidial, solitary, erumpent, globose to pulvinate, dark brown, 200–450 μm diam., exuding a brown conidial mass. ***Conidiophores*** indistinct, usually reduced to conidiogenous cells. ***Conidiogenous cells*** hyaline, smooth, pyriform base with long neck, straight or slightly curved, unbranched, phialidic, 10.5–44 × 1–2.5 μm. ***Conidia*** holoblastic, aseptate, hyaline, smooth, multi-guttulate, ellipsoidal, straight, (10.5–)11.5–13.5(–15) × 4.5–5(–5.5) μm (n = 50), L/W = 2.1–3.3.

##### Culture characteristics.

***Colonies*** on PDA erumpent, spreading, with flocculent aerial mycelia and undulating margin, forming concentric rings, forming brown vinaceous center area and smoke gray to pale purplish gray outer area, reaching 90 mm diam. after 1 week at 25 °C, forming dark brown conidiomata with brown conidial masses. ***Colonies*** on MEA flat, spreading, with moderate aerial mycelia and undulating margin, forming brown circular center area and buff outer area, reaching 70 mm diam. after 2 weeks at 25 °C, sterile. ***Colonies*** on SNA flat, spreading, with sparse aerial mycelia and undulating margin, white to smoke gray, reaching 70 mm diam. after 2 weeks at 25 °C, forming dark brown conidiomata with brown conidial masses.

##### Additional material examined.

China • Anhui Province, Hefei City, Shushan District, Dashushan Forest Park, on diseased leaves of *Quercusaliena*, 2 November 2019, Dan-ran Bian (culture CFCC 54375).

##### Distribution.

China, Anhui Province.

##### Ecology.

Associated with leaf spot disease of *Quercusaliena*.

##### Notes.

Two isolates from leaf spots of *Quercusaliena* clustered into a well-supported clade here newly described as *Pyrisporaquercicola*, which represents the fourth species of the genus *Pyrispora* and the family *Pyrisporaceae* (Fig. 1). *Pyrisporaquercicola* is similar to *Py.castaneae* in conidial shape and size (Jiang et al. 2021a). However, they can be distinguished by the host association and the molecular phylogeny (Fig. 1).

## ﻿Discussion

The classification of *Diaporthales* has undergone significant revisions over time, evolving from a system primarily based on teleomorph characteristics to a more comprehensive approach that integrates teleomorph, anamorph, and molecular phylogenetic data derived from ITS, LSU, *RPB2*, and *TEF1-α* gene sequences (Barr 1978; Castlebury et al. 2002; Cheewangkoon et al. 2010; Senanayake et al. 2016, 2017a, b, 2018; Fan et al. 2018; Hyde et al. 2020; Hongsanan et al. 2023). As demonstrated in recent taxonomic work, this integrated approach has successfully established a well-resolved classification system comprising 35 families (Zhang et al. 2025). In this study, we examined new specimens of *Pseudoplagiostomataceae* and *Pyrisporaceae* associated with leaf spots on *Fagaceae* hosts in China. Our findings led to the description of four novel species: *Ps.fagaceaearum*, *Ps.neocastanopsidis*, *Ps.quercus*, and *Py.quercicola*. Additionally, we propose the transfer of *Ps.humilis* and *Ps.myracrodruonis* to the genus *Pyrispora* based on their unthickened conidial walls, a taxonomic revision strongly supported by phylogenetic evidence. Furthermore, we synonymized *Neoplagiostoma* with *Pyrispora*, *N.castaneae* and *Ps.castaneae* with *Py.castaneae*, *Ps.ilicis* with *Ps.wuyishanense*, and *Ps.diaoluoshanense* with *Ps.mangiferae* due to their identical morphological features and molecular phylogeny.

Members of *Diaporthales* generally exhibit strong congruence between morphological characteristics and phylogenetic relationships, particularly within several well-defined groups such as *Cytosporaceae*, *Diaporthaceae*, and *Gnomoniaceae* (Senanayake et al. 2017a, 2018; Fan et al. 2020; Jiang et al. 2020). Furthermore, several families with distinctive morphological features have been firmly established in distinct phylogenetic positions, including *Asterosporiaceae*, *Coryneaceae*, *Erythrogloeaceae*, *Mastigosporellaceae*, and *Synnemasporellaceae* (Voglmayr and Jaklitsch 2014; Senanayake et al. 2017a, 2018; Jiang et al. 2024). Nevertheless, certain families demonstrate morphological convergence despite occupying distinct phylogenetic positions, as exemplified by *Juglanconidaceae*, *Pseudomelanconidaceae*, and *Melanconidaceae* (Voglmayr et al. 2012, 2017; Fan et al. 2018; Jiang et al. 2020). These observations highlight the necessity for expanded specimen collection, particularly from underrepresented families, to enhance our understanding of evolutionary patterns and diversification strategies within *Diaporthales*.

Recent studies have established *Pseudoplagiostoma* as an emerging fungal genus pathogenic to trees, primarily associated with leaf spot diseases (Suwannarach et al. 2016; Wang et al. 2016; Silva et al. 2023; Zhang et al. 2023; Haituk et al. 2024; Wu et al. 2024). The formation of appressoria by *Ps.jasmini* during infection of *Jasminumgrandiflorum* leaves has been documented, revealing a morphological feature characteristic of pathogenic fungi like *Colletotrichum* (Gomdola et al. 2023). However, *Pseudoplagiostoma* species appear less prevalent than their *Colletotrichum* counterparts, potentially due to their specialized host adaptation. As presented in Suppl. material 1, nearly all *Pseudoplagiostoma* species exhibit a strict association with tree hosts, which likely limits both their geographical distribution and evolutionary diversification.

Given the remarkable species diversity of *Fagaceae* hosts in China, which includes seven genera, *Quercus* exhibits a broad distribution, while *Castaneamollissima* is widely cultivated for its economic significance. Future research is likely to uncover additional species of *Pseudoplagiostomataceae* and *Pyrisporaceae*.

## Supplementary Material

XML Treatment for Pseudoplagiostoma fagacearum

XML Treatment for Pseudoplagiostoma jianfenglingense

XML Treatment for Pseudoplagiostoma mangiferae

XML Treatment for Pseudoplagiostoma neocastanopsidis

XML Treatment for Pseudoplagiostoma quercus

XML Treatment for Pseudoplagiostoma wuyishanense

XML Treatment for Pyrispora

XML Treatment for Pyrispora castaneae

XML Treatment for Pyrispora humilis

XML Treatment for Pyrispora myracrodruonis

XML Treatment for Pyrispora quercicola

## ﻿Additional information

### Adherence to national and international regulations

All the fungal strains used in this study have been legally obtained, respecting the Convention on Biological Diversity (Rio Convention).

### Author ORCIDs

Ning Jiang https://orcid.org/0000-0002-9656-8500

Han Xue https://orcid.org/0000-0003-0414-6237

Yong Li https://orcid.org/0000-0002-4406-1329

### Data availability

All of the data that support the findings of this study are available in the main text or Supplementary Information.

## Supplementary materials

Supplementary material 1 Species of *Pseudoplagiostoma* and *Pyrispora* and their Genbank accession numbers Data type pdf https://binary.pensoft.net/file/1335573This dataset is made available under the Open Database License (http://opendatacommons.org/licenses/odbl/1.0/). The Open Database License (ODbL) is a license agreement intended to allow users to freely share, modify, and use this Dataset while maintaining this same freedom for others, provided that the original source and author(s) are credited.Ning Jiang
