# Supplementary material for: ﻿Species diversity of Pseudoplagiostoma and Pyrispora (Diaporthales) from Fagaceae hosts in China
Source: IMA Fungus. 2025 May 23;16:e153782. doi: 10.3897/imafungus.16.153782 (PMC12125602; doi:10.3897/imafungus.16.153782)
Supplement: Supplementary material 1 — Species of Pseudoplagiostoma and Pyrispora and their Genbank accession numbers [file imafungus-16-e153782-s001.pdf]

Table S1 Species of *Pseudoplagiostoma* and *Pyrispora* and their Genbank accession numbers.

Species	Strains	Host	Country	GenBank Accession Numbers					References
				ITS	LSU	*TEF1-α*	*TUB2*	*RPB2*	
Pseudoplagiostoma alsophilae	SAUCC WZ0451*	*Alsophila spinulosa*	China	OP810625	OP810631	OP828580	OP828586	OP828578	Zhang et al. 2023
Ps. alsophilae	SAUCC WZ0152	*Alsophila spinulosa*	China	OP810626	OP810632	OP828581	OP828587	OP828579	Zhang et al. 2023
Ps. altingiae	CGMCC 3.28244*	*Altingia chinensis*	China	PQ338993	PQ339638	PQ358326	PQ358352	PQ367345	Zhang et al. 2025
Ps. altingiae	CGMCC 3.28245	*Altingia chinensis*	China	PQ338992	PQ339637	PQ358325	PQ358351	PQ367344	Zhang et al. 2025
Ps. altingiae	CGMCC 3.28246	*Altingia chinensis*	China	PQ338991	PQ339636	PQ358324	PQ358350	PQ367343	Zhang et al. 2025
Ps. altingiae	CGMCC 3.28242	*Schima superba*	China	PP101224	PP355149	PP379646	PP379689	PP379569	Zhang et al. 2025
Ps. bambusae	CGMCC 3.25363	*Acer buergerianum*	China	OR623171	OR623238	OR637439	OR637423	OR637431	Zhang et al. 2023
Ps. bambusae	CGMCC 3.25364	*Acer buergerianum*	China	OR623172	OR623239	OR637440	OR637424	OR637432	Zhang et al. 2023
Ps. bambusae	SAUCC 1206-4*	Bambusoideae sp.	China	OP810629	OP810635	OP828584	OP828590	NA	Zhang et al. 2023
Ps. bambusae	SAUCC 1206-6	Bambusoideae sp.	China	OP810630	OP810636	OP828585	OP828591	NA	Zhang et al. 2023
Ps. castanopsidis	CGMCC 3.28235*	*Castanopsis* sp.	China	PP446505	PP455436	PP453694	PP453702	PP453711	Zhang et al. 2025

Species	Strains	Host	Country	GenBank Accession Numbers					References
				ITS	LSU	*TEF1-α*	*TUB2*	*RPB2*	
Ps. castanopsidis	SAUCC 3969-1	*Castanopsis* sp.	China	PP101225	PP355150	PP379647	PP379690	PP379570	Zhang et al. 2025
Ps. corymbiae	CBS 132529*	*Corymbia* sp.	Australia	JX069861	JX069845	NA	NA	NA	Crous et al. 2012c
Ps. corymbiicola	CBS 145052*	*Corymbia citriodora*	Australia	MK047425	MK047476	MK047558	MK047577	NA	Crous et al. 2018
Ps. dipterocarpi	TBRC 1895*	*Dipterocarpus tuberculatus*	Thailand	KR994682	KR994683	NA	NA	NA	Suwannarach et al. 2016
Ps. dipterocarpicola	MFLUCC 21-0114	*Dipterocarpus* sp.	Thailand	OM228843	OM228841	OM219628	OM219637	NA	Tang et al. 2022
Ps. dipterocarpicola	MFLUCC 21-0142*	*Dipterocarpus* sp.	Thailand	OM228844	OM228842	OM219629	OM219638	NA	Tang et al. 2022
Ps. engelhardiae	CGMCC 3.28247*	*Engelhardia roxburghiana*	China	PQ338989	PQ339634	PQ358322	PQ358348	PQ367341	Zhang et al. 2025
Ps. engelhardiae	SAUCC 3185B–1	*Engelhardia roxburghiana*	China	PQ338990	PQ339635	PQ358323	PQ358349	PQ367342	Zhang et al. 2025
Ps. eucalypti	CPC 14161	*Eucalyptus camaldulensis*	Vietnam	GU973510	GU973604	GU973540	GU973573	NA	Cheewangkoon et al. 2010
Ps. eucalypti	CBS 124807*	*Eucalyptus urophylla*	Venezuela	GU973512	GU973606	GU973542	GU973575	NA	Cheewangkoon et al. 2010
Ps. eucalypti	SAUCC 4780	Eucalyptus robusta	China	PP101200	PP355146	PP379643	PP379686	PP379566	Zhang et al. 2025

Species	Strains	Host	Country	GenBank Accession Numbers					References
				ITS	LSU	*TEF1-α*	*TUB2*	*RPB2*	
Ps. fafuense	CGMCC 3.25357*	*Cinnamomum camphora*	China	OR623165	OR623232	OR637433	OR637417	OR637425	Mu et al. 2024b
Ps. fafuense	CGMCC 3.25358	*Cinnamomum camphora*	China	OR623166	OR623233	OR637434	OR637418	OR637426	Mu et al. 2024b
Ps. fagacearum	CFCC 54425*	*Quercus engleriana*	China	OK339766	OK339795	OK358605	OK358617	NA	In this study
Ps. fagacearum	CFCC 54446	*Quercus engleriana*	China	OK339767	OK339796	OK358606	OK358618	NA	In this study
Ps. fagacearum	CFCC 54410	*Quercus engleriana*	China	OK339768	OK339797	OK358607	OK358619	NA	In this study
Ps. fagacearum	CFCC 54449	*Castanopsis choboensis*	China	OK339769	OK339798	OK358608	OK358620	NA	In this study
Ps. fagacearum	CFCC 54393	*Cyclobalanopsis patelliformis*	China	OK339770	OK339799	OK358609	OK358621	NA	In this study
Ps. fargesiae	CGMCC 3.28252*	*Fargesia spathacea*	China	PQ338987	PQ339632	PQ358320	PQ358346	PQ367339	Zhang et al. 2025
Ps. fargesiae	SAUCC 5577-1	*Fargesia spathacea*	China	PQ338988	PQ339633	PQ358321	PQ358347	PQ367340	Zhang et al. 2025
Ps. fssistigmatis	CGMCC 3.28261*	*Fissistigma oldhamii*	China	PP446502	PP355144	PP379641	PP379684	PP379565	Zhang et al. 2025
Ps. fssistigmatis	SAUCC 5568-1	*Fissistigma oldhamii*	China	PP446501	PP455433	PP453699	PP453700	PP453709	Zhang et al. 2025
Ps. ilicis	CGMCC 3.25359	*Ilex chinensis*	China	OR623167	OR623234	OR637435	OR637419	OR637427	Mu et al. 2024b
Ps. ilicis	CGMCC 3.25360*	*Ilex chinensis*	China	OR623168	OR623235	OR637436	OR637420	OR637428	Mu et al. 2024b
Ps. indicum	CGMCC 3.28238*	*Mangifera indica*	China	PP101202	PP355156	PP379652	PP379696	PQ367346	Zhang et al. 2025
Ps. indicum	SAUCC 3269	*Mangifera indica*	China	PP446500	PP455438	PP453695	PP453704	PQ367347	Zhang et al. 2025

Species	Strains	Host	Country	GenBank Accession Numbers					References
				ITS	LSU	*TEF1-α*	*TUB2*	*RPB2*	
Ps. inthanonense	MFLU 23-0345*	Unknown trees	Thailand	NR_191298	NG_243399	OR650831	OR611920	OR611921	Silva et al. 2023
Ps. jasmini	MFLUCC 23-0044*	*Jasminum grandiflorum*	Thailand	OQ786078	OQ786079	OQ850145	OQ850148	NA	Gomdola et al. 2023
Ps. jianfenglingense	CGMCC 3.28254*	Unknown leaves	China	PQ338983	PQ339628	PQ358316	PQ358342	PQ367335	Zhang et al. 2025
Ps. jianfenglingense	SAUCC 3971	Unknown leaves	China	PQ338984	PQ339629	PQ358317	PQ358343	PQ367336	Zhang et al. 2025
Ps. jianfenglingense	CFCC 54396	*Castanopsis patelliformis*	China	OK339764	OK339793	OK358603	OK358615	NA	In this study
Ps. jianfenglingense	CFCC 55894	*Castanopsis patelliformis*	China	OK339765	OK339794	OK358604	OK358616	NA	In this study
Ps. jinghongense	CGMCC 3.28265*	*Machilus nanmu*	China	PP101237	PP355169	PP379665	PP379709	PP379586	Zhang et al. 2025
Ps. jinghongense	SAUCC 2287-5	*Machilus nanmu*	China	PP446509	PP455441	PP453698	PP453707	PP453715	Zhang et al. 2025
Ps. kunmingense	CGMCC 3.28240*	*Machilus nanmu*	China	PP101222	PP355145	PP379642	PP379685	PP379588	Zhang et al. 2025
Ps. kunmingense	SAUCC 5402-2	*Machilus nanmu*	China	PP446503	PP455434	PP453692	PP453708	PP453716	Zhang et al. 2025
Ps. ligustri	CGMCC 3.28241*	*Ligustrum ovalifolium*	China	PP101226	PP355152	PQ358327	PP379692	PP379572	Zhang et al. 2025
Ps. ligustri	SAUCC 3790-1	*Ligustrum ovalifolium*	China	PP446506	PP455437	PQ358328	PP453703	PP453712	Zhang et al. 2025

Species	Strains	Host	Country	GenBank Accession Numbers					References
				ITS	LSU	*TEF1-α*	*TUB2*	*RPB2*	
Ps. machili	SAUCC BW0233*	*Machilus nanmu*	China	OP810627	OP810633	OP828582	OP828588	NA	Zhang et al. 2023
Ps. machili	SAUCC BW0221	*Machilus nanmu*	China	OP810628	OP810634	OP828583	OP828589	NA	Zhang et al. 2023
Ps. machili-nanmu	CGMCC 3.28253*	*Machilus nanmu*	China	PQ338985	PQ339630	PQ358318	PQ358344	PQ367337	Zhang et al. 2025
Ps. machili-nanmu	SAUCC 1611	*Machilus nanmu*	China	PQ338986	PQ339631	PQ358319	PQ358345	PQ367338	Zhang et al. 2025
Ps. mangiferae	KUMCC 18-0179*	*Mangifera* sp.	China	MK084824	MK084825	NA	NA	NA	Phookamsak et al. 2019
Ps. mangiferae (syn. *Ps. diaoluoshanense*)	CGMCC 3.28236*	*Mangifera indica*	China	PP101232	PP355161	PP379657	PP379701	PP379578	Zhang et al. 2025
Ps. mangiferae (syn. *Ps. diaoluoshanense*)	SAUCC 2892	*Mangifera indica*	China	PP446508	PP455440	PP453697	PP453706	PP453714	Zhang et al. 2025
Ps. neocastanopsidis	CFCC 54447*	*Castanopsis carlesii*	China	OK339762	OK339791	OK358601	OK358613	NA	In this study
Ps. neocastanopsidis	CFCC 52809	*Castanopsis carlesii*	China	OK339763	OK339792	OK358602	OK358614	NA	In this study
Ps. oldii	CBS 124808*	*Eucalyptus camaldulensis*	Australia	GU973534	GU973609	GU973564	GU993862	NA	Cheewangkoon et al. 2010

Species	Strains	Host	Country	GenBank Accession Numbers					References
				ITS	LSU	*TEF1-α*	*TUB2*	*RPB2*	
Ps. oldii	CBS 115722	*Eucalyptus camaldulensis*	Australia	GU973535	GU973610	GU973565	GU993864	NA	Cheewangkoon et al. 2010
Ps. perseae	BCRC FU31388*	*Persea americana*	China	MT233359	MT233373	NA	MT251143	NA	Wu et al. 2024
Ps. perseae	BCRC FU31389	*Persea americana*	China	MT233360	MT233374	NA	MT251144	NA	Wu et al. 2024
Ps. quercus	CFCC 55232*	*Quercus aliena*	China	OK339771	OK339800	OK358610	OK358622	NA	In this study
Ps. quercus	CFCC 55192	*Quercus aliena*	China	OK339772	OK339801	OK358611	OK358623	NA	In this study
Ps. quercus	CFCC 55262	*Quercus variabilis*	China	OK339773	OK339802	OK358612	OK358624	NA	In this study
Ps. sanmingense	CGMCC 3.25361	*Photinia glabra*	China	OR623169	OR623236	OR637437	OR637421	OR637429	Mu et al. 2024b
Ps. sanmingense	CGMCC 3.25362*	*Quercus glauca*	China	OR623170	OR623237	OR637438	OR637422	OR637430	Mu et al. 2024b
Ps. tonkinense	CGMCC 3.28243*	*Exbucklandia tonkinensis*	China	PP101229	PP355158	PP379654	PP379698	PP379575	Zhang et al. 2025
Ps. tonkinense	SAUCC 3186-2	*Exbucklandia tonkinensis*	China	PP446507	PP455439	PP453696	PP453705	PP453713	Zhang et al. 2025
Ps. variabile	CBS 113067*	*Eucalyptus globulus*	Uruguay	GU973536	GU973611	GU973566	GU993863	NA	Cheewangkoon et al. 2010
Ps. wuyishanense	CGMCC 3.25367	Unknown trees	China	PP658312	NA	NA	PP665723	PP665719	Mu et al. 2024a
Ps. wuyishanense	CGMCC 3.25368 *	Unknown trees	China	PP658313	NA	NA	PP665724	PP665720	Mu et al. 2024a
Ps. wuyishanense (syn. *Ps. ilicis*)	CGMCC 3.25359	*Ilex chinensis*	China	OR623167	OR623234	OR637435	OR637419	OR637427	Mu et al. 2024b

Species	Strains	Host	Country	GenBank Accession Numbers					References
				ITS	LSU	*TEF1-α*	*TUB2*	*RPB2*	
Ps. wuyishanense (syn. *Ps. ilicis*)	CGMCC 3.25360	*Ilex chinensis*	China	OR623168	OR623235	OR637436	OR637420	OR637428	Mu et al. 2024b
Pyrispora castaneae	CFCC 54349 *	*Castanea mollissima*	China	MW208108	MW208105	MW227340	NA	MW218535	Jiang et al. 2021a
Py. castaneae	CFCC 54350	*Castanea mollissima*	China	MW208109	MW208106	MW227341	NA	MW218536	Jiang et al. 2021a
Py. castaneae	SAUCC my0162	*Castanea mollissima*	China	MZ156982	MZ156985	MZ220321	MZ220325	MZ220323	Mu et al. 2022
Py. castaneae	SAUCC my0523	*Castanea mollissima*	China	MZ156983	MZ156986	MZ220322	MZ220326	MZ220324	Mu et al. 2022
Py. humilis (syn. *Ps. humilis*)	CCUB 5837*	*Anacardium humile*	Brazil	OR452735	NA	OR540677	OR420725	NA	Magalhães et al. 2024
Py. humilis (syn. *Ps. humilis*)	CCUB 5838	*Anacardium humile*	Brazil	OR452736	NA	OR540678	OR420726	NA	Magalhães et al. 2024
Py. myracrodruonis (syn. *Ps. myracrodruonis*)	URM 7799*	*Astronium urundeuva*	Brazil	MG870421	MK982151	MK982557	MN019566	MK977723	Bezerra et al. 2019
Py. myracrodruonis (syn. *Ps. myracrodruonis*)	URM 8123	*Astronium urundeuva*	Brazil	MK982150	MK982152	MK982558	MN019567	MK977724	Bezerra et al. 2019
Py. quercicola	CFCC 54868*	*Quercus aliena*	China	OK339774	OK339803	OK358589	NA	OK358591	In this study
Py. quercicola	CFCC 54375	*Quercus aliena*	China	OK339775	OK339804	OK358590	NA	OK358592	In this study

Note. Ex-type strains are marked with *, and NA means not available.
